# Risk for Infection with Highly Pathogenic Avian Influenza Virus (H5N1) in Backyard Chickens, Bangladesh

**DOI:** 10.3201/eid1512.090643

**Published:** 2009-12

**Authors:** Paritosh K. Biswas, Jens P. Christensen, Syed S.U. Ahmed, Ashutosh Das, Mohammad H. Rahman, Himel Barua, Mohammad Giasuddin, Abu S.M.A. Hannan, Mohammad A. Habib, Nitish C. Debnath

**Affiliations:** Chittagong Veterinary and Animal Sciences University, Chittagong, Bangladesh (P.K. Biswas, S.S.U. Ahmed, A. Das, M.H. Rahman, H. Barua, N.C. Debnath); Copenhagen University, Copenhagen, Denmark (J.P. Christensen); Bangladesh Livestock Research Institute, Dhaka, Bangladesh (M. Giasuddin); Department of Livestock Services, Dhaka (A.S.M.A. Hannan, M.A. Habib)

**Keywords:** Highly pathogenic avian influenza, H5N1, backyard chickens, risk factors, influenza, viruses, research

## Abstract

To evaluate risk factors for infection with highly pathogenic avian influenza A virus (H5N1) in backyard chickens in Bangladesh, we conducted a matched case–control study. We enrolled 25 case farms (cases March–November 2007) and 75 control farms (June–November 2007). We used a questionnaire to collect farm data, which were analyzed by matched-pair analysis and multivariate conditional logistic regression. Factors independently associated were offering slaughter remnants of purchased chickens to backyard chickens (odds ratio [OR] 13.29, 95% confidence interval [CI] 1.34–131.98, p = 0.027), having a nearby water body (OR 5.27, 95% CI 1.24–22.34, p = 0.024), and having contact with pigeons (OR 4.47, 95% CI 1.14–17.50, p = 0.032). Separating chickens and ducks at night was protective (OR 0.06, 95% CI 0.01–0.45, p = 0.006). Reducing these risks and taking protective measures might reduce the risk for influenza (H5N1) infection in backyard chickens.

## Abstract 2

Risk factors were feeding slaughter remnants of purchased chickens, having a body of water nearby, and contact with pigeons.

Highly pathogenic avian influenza (HPAI) A virus (H5N1) is a deadly zoonotic pathogen; from 1997 through 2008, a total of 413 human cases were reported in 15 countries, and 256 persons died (*1*). By March 2008, the virus had been identified in birds in 61 countries (*2*). The persistence of the virus in poultry over a wide geographic area strengthens the hypothesis that a mutant virus might evolve and initiate a human pandemic. To reduce this threat, every country should have a surveillance system for detecting the virus in poultry, including backyard flocks.

Worldwide, poultry production has recently undergone rapid change, including the introduction of intensive production, new breeds, improved biosecurity, and preventive health measures. In developing countries, however, adoption of this type of production has been limited because of the costs of infrastructures to maintain biosecurity for birds, quality hybrid chicks, balanced feed, biologics, and quality veterinary care (*3*). Up to 80% of the poultry in Africa and Asia are kept in backyard-type systems (*3*,*4*), and these birds represent a substantial economic resource for impoverished rural populations.

In Bangladesh, ≈89% of rural households have backyard poultry (*5*), and many households keep chickens and ducks on the same property (*6*). In the absence of fences or other barriers, backyard chickens roam freely from one property to another. Because backyard chickens are reared in such free-range systems, they are more vulnerable to the HPAI (H5N1) virus infection; and, if they become infected, they can transmit the virus to domestic ducks, in which the virus can perpetuate (*7*–*9*) and infect more backyard chickens. This cycle of virus transmission between backyard chickens and ducks would continue until intercepted. HPAI (H5N1) virus in backyard chickens also poses a serious threat to public health because of the frequent and close contacts between poultry and humans. Little has been published about the risk factors associated with HPAI (H5N1) virus infection in backyard chickens in any parts of the world, and to our knowledge, nothing has been published about the risk factors in Bangladesh. Because understanding the risk factors for the virus in backyard chickens and preventive measures might slow or prevent the spread of the virus, we conducted a case-control study to determine the risk factors for HPAI (H5N1) virus infection in backyard chickens in Bangladesh.

## Materials and Methods

### Study Population and Case Definition

Bangladesh is composed of 4,500 unions (local government units that comprise several villages) and 90,500 villages (*10*). Of the total poultry population in the country (≈222 million birds), 50% are backyard poultry, predominantly indigenous (nondescriptive) chickens and mostly reared in free-range systems on the homesteads in these villages (*10*). In Bangladesh, villagers sometime rear Fayoumi and Sonali (a cross-bred F1 generation of Fayoumi [female] and Rhode Island Red [male]) chickens in a semi-scavenging system (*11*–*13*) and occasionally in intensive systems. All 25 HPAI outbreaks recorded in indigenous (n = 20 farms), Fayoumi (n = 2), and Sonali (n = 5) chickens in village areas in Bangladesh by November 17, 2007, were considered outbreaks in backyard chickens, and the farms were enrolled in our study as case backyard farms. By date of onset of clinical signs, the first outbreak of HPAI in backyard chickens was recorded on March 22, 2007, the date on which Bangladesh was officially declared HPAI (H5N1) virus infected. In 2007, the numbers of backyard farms infected were 1 farm in March, 3 in April, 7 in May, 7 in June, 2 in July, 1 in September, 3 in October, and 1 in November.

A case backyard farm was defined as one that had a high chicken mortality rate and on which influenza virus A subtype H5 was detected from tracheal samples of 2 chickens by reverse transcription–PCR (RT-PCR) using a primer set hemagglutinin (HA) oligo 5′ (5′-ACACATGCYCARGACATACT-3′) and HA oligo 3′ (5′-CTYTGRTTYAGTGTTGATGT-3′), as described by Lee et al. (*14*). Testing was done at the National Reference Laboratory for Avian Influenza in Bangladesh. Case reporting of HPAI (H5N1) in chickens in Bangladesh and detailed laboratory diagnosis, including diagnostic reconfirmation from the Veterinary Laboratory Agency in the United Kingdom, has been described (*15*).

For each case farm, we selected and enrolled 3 control backyard farms, each of which was within 1–10 km of a case farm. Each unaffected village in this zone of selection was assigned a unique number, and 2 were randomly selected by lottery. One villager from each selected village was asked to give 10 names of the backyard farm owners in the village who had reared village chickens for >1 year. These 10 names with distinct numbers were used as the sample frame for the village. From the sample frame of the first selected village, we randomly chose 2 backyard farm owners who had adult chickens (>6 months of age) and chicks (<1 month of age) and whose chickens had not died during the clinical phase of HPAI on the case farm. Likewise, 1 backyard farm owner was selected from the sample of the second village. To find control farms with Sonali or Fayoumi chickens, the same (1–10 km) zone of selection was used, but the names of the farm owners were drawn from the local upazila (a lower administrative unit in Bangladesh) veterinary office and used as the sample from which to randomly select 3 farms. Because biologics were scarce, serologic testing to confirm the noninfected status of the control farms was not attempted.

Global positioning system coordinates from the case and control farms were collected during farm visits and entered into a digitized map of Bangladesh. A geographic information system program (Arc View 9.1; Environmental System Research Institute, Redlands, CA, USA) was used (Figure).

**Figure:**
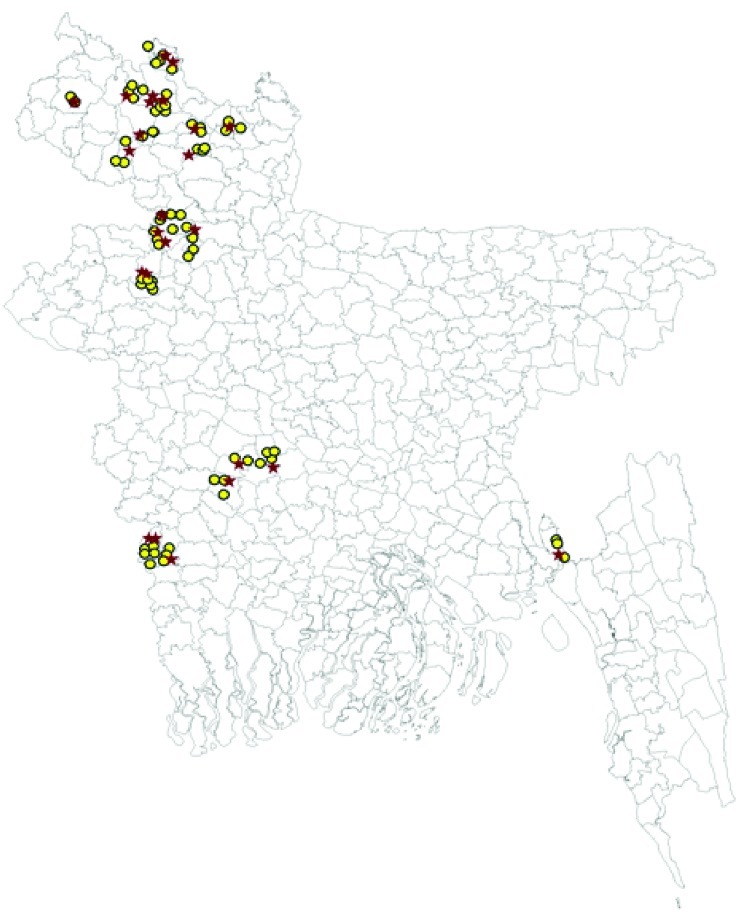
Locations of 25 backyard farms where outbreaks of highly pathogenic avian influenza A virus (H5N1) infection occurred during March–November 2007 (red stars) and 75 control backyard farms (yellow circles), Bangladesh.

### Data Collection and Survey Method

A questionnaire designed for this study was pretested at 5 case farms. The questionnaire was then modified according to new variables encountered during pretesting. In the final questionnaire, 59 variables were surveyed (spreadsheet available from P.K.B.). The questionnaire was then administered on the case and control farms by 2 veterinarians trained to administer questionnaires; they interviewed farm owners or, if owners were absent, any adult family members. Variables collected addressed geographic location, stock information, flock health history, and overall farm management. All interviews were conducted in Bengali, the only spoken language in the study area, during June–November 2007.

### Statistical Analysis

The collected data were entered into a spread sheet program (Excel; Microsoft, Redmond, WA, USA) and transferred into Epi Info 2000 (Centers for Disease Control and Prevention, Atlanta, GA, USA) for analysis. To estimate the strength and statistical significance of associations between risk factors and HPAI (H5N1) virus infection, we used the Mantel-Haenszel matched-pair analysis (McNemar) test. An association was considered significant if 2-sided tests of significance had a p value <0.05. To examine independence of effects, we conducted multivariate conditional logistic regression using the conditional logistic regression (CLogit) function in Stata 9.0 for Windows (Stata Corp., College Station, TX, USA). Any variables with p<0.2 after matched-pair analysis were included in the initial model. A backward stepwise variable–selection strategy was used to construct a final model with a significance level of p<0.05.

## Results

### Population Statistics

Indigenous, Fayoumi, and Sonali chickens were raised on 20, 2, and 3 backyard farms, respectively. Median number (and range) of indigenous chickens was 24 (3–88) on case farms and 14 (5–50) on control farms and of Fayoumi/Sonali chickens was 950 (125–1,970) on case farms and 2,200 (1,500–3,500) on control farms. Chickens of various ages (mean 35.7 weeks [range 3.5–130]) were raised on 10 of the case farms, but precise ages of chickens on 15 case farms were not provided by the owners; 6 said they had only adult chickens, and 9 said they had adult chickens and young chicks. Adult and young indigenous chickens were raised on the 60 control farms.

### Matched-Pair Analysis

The results of matched-pair analysis (Table 1) showed that offering slaughter remnants of purchased chickens to backyard chickens (within 21 days of the onset clinical signs in case farms) had the strongest point estimate of effect (matched odds ratio [OR] 22.1) and high statistical significance (p<0.001) despite wide 95% confidence intervals (CIs) of 2.7–177.7. Other factors positively associated with case farms were migratory birds around a farm (OR 7.5, 95% CI 1.5–38.7, p = 0.010), rodents on the farm (OR 5.8, 95% CI 2.0–16.8, p = 0.001), contact with pigeons (OR 5.5, 95% CI 1.9–16.0, p = 0.001), and a nearby body of water (OR 3.7, 95% CI 1.5–9.5, p = 0.004). Protective factors (OR <1) for case backyard farms were placing chickens and ducks in different shelters at night (OR 0.1, 95% CI 0.1–0.5, p = 0.001) and having a commercial farm within 0.5 km (OR 0.3, 95% CI 0.1–0.9, p = 0.028).

**Table 1:** Matched-pair analysis of potential risk factors for highly pathogenic avian influenza virus (H5N1) in backyard chickens, Bangladesh, 2007

Risk factor	Case farms (n = 25), no. (%)	Control farms (n = 75), no. (%)	Matched OR (95% CI)*	p value
Farm <5 km from nearest veterinary hospital	15 (60.0)	41 (54.7)	1.3 (0.5–3.2)	0.636
Nearby (<0.1 km) body of water	16 (64.0)	23 (30.7)	3.7 (1.5– 9.5)	0.004
Farm <0.5 km from larger body of water	15 (60.0)	50 (66.7)	0.7 (0.3–1.9)	0.543
Commercial farm within 0.5 km	9 (36.0)	44 (58.7)	0.3 (0.1–0.9)	0.028
Migratory birds seen around farm	6 (24.0)	5 (6.7)	7.5 (1.5–38.7)	0.010
Local live bird market within <5-km radius	24 (96.0)	73 (97.3)	0.7 (0.1–7.4)	0.747
Farm <1 km from live bird market	17 (68.0)	50 (66.7)	1.1 (0.4–3.0)	0.895
Contact with ducks	22 (88.0)	55 (73.3)	4.0 (0.8–20.1)	0.062
Contact with pigeons	16 (64.0)	21 (28.0)	5.5 (1.9–16.0)	0.001
Presence of rodents	12 (48.0)	9 (12.0)	5.8 (2.0–16.8)	0.001
Chickens and ducks on the same farm	11 (44.0)	40 (53.3)	0.7 (0.3–1.7)	0.415
Chickens and ducks in different night shelters	2 (8.0)	31 (41.3)	0.1 (0.1–0.5)	0.001
Frequent (≈1×/wk) cleaning of shelter	18 (72.0)	44 (58.7)	1.8 (0.7–4.9)	0.221
No disinfection in shelter	4 (16.0)	10 (13.3)	3.0 (0.2–48.0)	0.448
Disposal of bird in open space	19 (76.0)	58 (77.3)	0.9 (0 .3– 3.2)	0.869
Recently purchased chickens brought in†	5 (20.0)	10 (13.3)	1.7 (0.5–5.7)	0.421
Offering slaughter remnants of purchased chickens†	8 (32.0)	2 (2.7)	22.1 (2.7–177.7)	0.000
Death of neighbor’s chickens	7 (28.0)	20 (26.7)	1.1 (0.4–2.8)	0.900
Source of chicks = own hatched	5 (20.0)	20 (26.7)	0.4 (0.1–2.0)	0.226

*Matched-pair analysis using McNemar (Mantel-Haenszel) test statistics. OR, odds ratio; CI, confidence interval. †Within 21 days of the onset clinical signs on case backyard farms.

### Multivariate Analysis

Eight variables with p<0.2 were considered for inclusion in the conditional logistic regression model to estimate independence of effects (Table 2). The final conditional logistic regression model identified 3 variables as independent risk factors for HPAI (H5N1) infection of backyard chickens in Bangladesh (Table 3). They were 1) offering slaughter remnants of purchased chickens to backyard chickens (within 21 days of the clinical onset of the disease) (OR 13.29, 95% CI 1.34–131.98), 2) having nearby body of water (OR 5.27, 95% CI 1.24–22.34), and 3) having contact with pigeons (OR 4.47, 95% CI 1.14–17.50)**.** The final model also identified a protective factor: placing chickens and ducks in different shelters at night (OR 0.06 95% CI 0.01–0.45).

**Table 2:** Initial results from multivariate analysis of potential risk factors for highly pathogenic avian influenza virus (H5N1) in backyard chickens, Bangladesh, 2007*

Risk factor	Odds ratio	95% Confidence interval	p value
Nearby (<0.1 km) body of water	3.64	0.82–16.18	0.089
Commercial farm within 0.5 km	3.57	0.34– 37.82	0.291
Migratory bird seen around farm	3.37	0.05–234.59	0.575
Contact with ducks	1.47	0.15–14.29	0.740
Contact with pigeons	7.64	1.00–58.48	0.050
Presence of rodents	7.94	0.89–72.61	0.067
Chickens and ducks in different night shelters	0.08	0.01–0.71	0.023
Offering slaughter remnants of purchased chickens†	9.02	0.77–105.79	0.080

*Conditional logistic regression; initial set with 8 variables entered; χ^2^ for likelihood ratio test = 43.85; p>0.001; no. observations = 100. †Within 21 days of the onset clinical signs in case backyard farms.

**Table 3:** Results of final model with potential risk factors for highly pathogenic avian influenza virus (H5N1) in backyard chickens, Bangladesh, 2007*

Risk factor	Odds ratio	95% Confidence interval	p value
Nearby (<0.1 km) body of water	5.27	(1.24–22.34)	0.024
Contact with pigeons	4.47	(1.14–17.50)	0.032
Chickens and ducks in different night shelters	0.06	(0.01–0.45)	0.006
Offering slaughter remnants of purchased chickens†	13.29	(1.34–131.99)	0.027

*Conditional logistic regression; final model with 4 variables entered; χ^2^ for likelihood ratio test = 38.82; p>0.001; no. observations = 100. †Within 21 days of the onset clinical signs in case backyard farms.

## Discussion

We used analytic epidemiologic techniques to unveil the possible risk factors associated with influenza (H5N1) infection for backyard chickens in Bangladesh so that effective risk management can be advocated. A few published reports quantify the risk factors for influenza (H5N1) infections in commercial chickens (*16*,*17*), but to our knowledge, analytic epidemiologic reports quantifying risk factors for backyard chickens are few, if any. The results of this study should contribute to the understanding of risk factors associated with influenza (H5N1) infections in backyard chickens in other developing countries, particularly in southern Asia.

Although only 1 case of influenza (H5N1) in a human has been reported in Bangladesh, the country’s poultry sector has been severely affected; by July 2009, a total of 325 outbreaks had been reported in chickens, 51 of which were in backyard chickens (www.mofl.gov.bd/daily_birdflu_report.pdf). Because of limited manpower, the country relies predominantly on passive surveillance to detect HPAI outbreaks in chickens. Thus, the possibility of unreported cases occurring in backyard chickens in some parts of the country cannot be ruled out. These hidden and unreported infections in backyard chickens can help perpetuate the virus, posing a serious challenge to eradication efforts. Strengthening active instead of passive surveillance and generating awareness at the rural level of risk factors for the HPAI (H5N1) virus infection in backyard chickens, and their management, might help reduce the virus load in poultry in the country.

Stalls with live poultry can be found at virtually every kitchen or village market in Bangladesh. At the local markets, villagers can sell their poultry to local persons or to poultry vendors, who buy poultry in bulk to sell at larger city markets. When villagers fear a disease outbreak, they start selling apparently healthy and even clinically diseased chickens. Diseased chickens are cheaper, encouraging other villagers to buy them for meat. They purchase live chickens and slaughter them at home. They then offer the slaughter remnants, inedible for humans, to their own backyard chickens, which scavenge and forage around the slaughter places. Such practice occurred at 8 (32%) case farms <21 days of the onset of the clinical signs (Table 1); this practice appears to be strongest risk factor for HPAI (H5N1) infection in backyard chickens in Bangladesh.

Another risk factor was domestic ducks, which are considered a “Trojan horse” for the HPAI (H5N1) virus (*7*–*9*). Their main feed sources are vegetation, small fish, amphibians, snails, oysters, and other crustaceans, found in and around water. The water bodies and their banks might become contaminated with the HPAI (H5N1) virus by virus-shedding ducks that congregate at these places. Backyard chickens might be exposed to the virus while sharing the same banks near the body of water, which could explain why a nearby body water appeared to be an independent risk factor.

The influence of 2 kinds of bodies of water on the HPAI outbreaks in backyard chickens was assessed by incorporating 2 variables: 1) presence of a nearby (<0.1 km) body of water and 2) distance <0.5 km from a larger body of water. The latter variable was meant for any larger water-logged paddy or open field, lagoon, marsh, river, lake, or canal where water and migratory birds live or take refuge. These bodies of water are sometimes shared by domestic ducks; but generally, ducks on backyard farms feed on nearby bodies of water, predominantly ponds made by the birds’ owners for household purposes or aquaculture. Secondarily, ducks roam in these ponds to collect feed. Presence of a larger body of water within 0.5 km of a backyard farm seems to have no causal association with the occurrence of HPAI in backyard chickens in Bangladesh, but the presence of a nearby pond might.

In 1997, domestic pigeons (*Columbia* spp.) were largely resistant to infection with an HPAI (H5N1) virus isolated from Hong Kong Special Administrative Region, People’s Republic of China (*18*); other studies showed that they appeared to be more resistant to infection than many other avian species (*19*,*20*). In Bangladesh, many backyard farmers rear chickens, ducks, pigeons, and sometimes other poultry in groups of mixed ages. Domestic pigeons are a major source of meat in Bangladesh, and not 1 pigeon in the country has been reported dead of influenza (H5N1) infection. The risk factor of contact with pigeons included 2 categories: the owners’ own domestic pigeons and neighbors’ visiting pigeons. In a complex of backyard farms in Bangladesh, pigeons are allowed to feed with other farm poultry; in addition, pigeons of 1 backyard farm frequently visit others for additional feed. Oronasal secretions and feces from sick, or dead, backyard chickens with HPAI, have a high virus titer, thereby polluting the farm. Pigeons’ feeding and behavior probably allows them to come in close contact with the secretions of the infected or dead chickens or with fomites, enabling them to transmit the virus mechanically. Nettles et al. (*21*) reported that pigeons and some wild birds—crows, mourning doves, vultures, and others—are not responsible for dissemination of influenza virus (H5N2) among poultry farms. However, in contrast to the findings of Nettles et al. (*21*), dead crows in different areas of Bangladesh were found to be positive for influenza virus (H5) (neuraminidase was not determined) (*22*). Because of the lack of evidence of mechanical transmission of influenza virus (H5N1) through pigeons in backyard chickens, the hypothesis that they are mechanical transmitters of influenza virus (H5N1) under the prevailing conditions of backyard chicken farms in Bangladesh cannot be confirmed without a thorough virologic study.

Some owners also offer supplementary feeds, predominantly cereals or their byproducts, to their chickens and ducks, usually in the evening when they are placed in the night shelters to protect them from predators. No domestic duck in Bangladesh has been reported dead of influenza virus (H5N1) infection. Placing chickens and ducks in separate night shelters appeared to be a protective factor. On the contrary, an association with influenza (H5N1) was found with rearing them on the same farm.

In developing countries, including Bangladesh, biosecurity enhancement, according to the Food and Agriculture Organization of the United Nations, poultry production system 4 is impossible to adopt. However, practical ways to minimize the risk factors identified in this study are possible in these countries, as follows. Chickens must not be fed remnants of slaughtered chickens that have been purchased from markets or mobile poultry vendors, and inedible portions thus produced must be disposed of hygienically. Villagers should not buy any obviously or apparently sick chickens, although they are cheaper, because these birds pose a serious threat to the villagers’ health and to the health of their backyard chickens. Enactment of laws with punitive measures for selling clinically sick chickens anywhere in the country and strict implementation of these laws are vital for limiting the spread of the virus from live bird markets to backyard chickens and vice versa. Individual backyard farm owners should be encouraged to rear only chickens or ducks; but if that is impractical, the owners should be advised to construct separate night shelters for ducks and chickens. Chickens should be limited or prevented from scavenging along the banks of bodies of water. During feeding, a family member can prevent pigeons from joining the flock; any remaining feed must be removed carefully.

Because backyard chickens are a vital economic resource in Bangladesh, backyard farmers cannot be prevented from rearing them. Therefore, avoidance of the risk factors identified in this study, and implementation of protective factors, might reduce the risk for influenza (H5N1) infection in backyard chickens in the country.
